# Evolution of plant genome architecture

**DOI:** 10.1186/s13059-016-0908-1

**Published:** 2016-03-01

**Authors:** Jonathan F. Wendel, Scott A. Jackson, Blake C. Meyers, Rod A. Wing

**Affiliations:** Department of Ecology, Evolution and Organismal Biology, Iowa State University, Ames, IA 50011 USA; Center for Applied Genetic Technologies, University of Georgia, Athens, GA 30602 USA; Donald Danforth Plant Science Center, 975 North Warson Road, St. Louis, MO 63132 USA; Division of Plant Sciences, University of Missouri–Columbia, 52 Agriculture Laboratory, Columbia, MO 65211 USA; Arizona Genomics Institute, School of Plant Sciences and Department of Ecology and Evolutionary Biology, Tucson, AZ 85750 USA; T.T. Chang Genetic Resource Center, International Rice Research Institute, Los Baños, Laguna Philippines

## Abstract

We have witnessed an explosion in our understanding of the evolution and structure of plant genomes in recent years. Here, we highlight three important emergent realizations: (1) that the evolutionary history of *all* plant genomes contains multiple, cyclical episodes of whole-genome doubling that were followed by myriad fractionation processes; (2) that the vast majority of the variation in genome size reflects the dynamics of proliferation and loss of lineage-specific transposable elements; and (3) that various classes of small RNAs help shape genomic architecture and function. We illustrate ways in which understanding these organism-level and molecular genetic processes can be used for crop plant improvement.

## Background

The readers of *Genome Biology* are likely to agree that we are living in a tremendously exciting time to be a biologist, perhaps one that in the future will be thought of as a ‘golden era’, replete with technological and conceptual breakthroughs. These breakthroughs are synergistic twins, of course, as novel analytical methods lead to applications that generate biological discoveries and hypotheses that are conceptually transformative. This synergy is particularly evident in the study of plant genome evolution, in which massively parallel sequencing approaches have revealed genomic diversity in exquisite detail, which has led to many insights into genome function and evolution. Our purpose in this short review is to highlight progress made in the understanding of plant genome evolution, with a focus on crop plants and on recent key insights. We highlight that modern plant genomes derive from processes set in motion by a history of repeated, episodic whole-genome doubling events, and that the extraordinary variation in genome size across plant species largely reflects differences in proliferation and survival of various classes and families of transposable elements (TEs), often in a lineage-specific manner. Furthermore, we discuss the connections between genomic architecture and small RNA function. As our review is mainly focused on crop plant genomes, we also discuss how plant genomics is relevant to crop improvement and food security.

## Whole-genome doubling: wash, rinse, repeat

One of the important realizations of the genomics era is that whole-genome doubling (WGD), or polyploidy, is far more prevalent in the evolutionary history of plants than previously recognized. Classic estimates based on comparative cytogenetic studies [1–3] and stomatal guard cell sizes [4] have indicated that chromosome doubling is common in many genera and families, with estimates of the frequency of polyploid ancestry ranging from 35 % to 70 %. Thus, polyploidy has long been appreciated as important in angiosperm diversification and as an active mode of speciation in many groups. Polyploidy leading to speciation can arise by several means [5], either within single individuals or following hybridization between closely related populations (autopolyploidy), or from interspecific or, more rarely, intergeneric hybridization events (allopolyploidy) [6].

### The ubiquity and cyclical nature of polyploidy

Genomic analyses over the past 15 years have demonstrated that *all* flowering plants are polyploid, and multiply so [7–9]. That is, the phylogenetic history of angiosperms abounds with WGD events, the most recent of which are superimposed on earlier duplications that took place early in angiosperm evolution, and before that on duplications that occurred at the root of the seed plants [7]. Our understanding of the cyclical nature of polyploidy was first suggested by analyses of expressed sequence tags (ESTs) in many different plant species (or genera). These analyses revealed ‘peaks’ of sequence similarity among genes within genomes representing multiple gene duplicates, whose collective existence and features suggest they traced to a common origin [10]. In many cases, several such peaks existed within individual genomes, which ostensibly reflects progressively more ancient WGD events. This emerging view of the canonical angiosperm genome as one that has experienced multiple episodic polyploidy events has been confirmed by recent genome sequencing efforts (Table 1). These studies revealed a widespread pattern of nested, intragenomic syntenies, often shared among close relatives but varying widely and in a lineage-specific fashion among different angiosperm groups. Therefore, we can rightfully replace the obsolete question ‘is this species polyploid?’ with the more appropriate ‘when did genome duplication occur and how many rounds of genome doubling have occurred in the history of this particular species?’

**Table 1:** Sequenced crop genomes with their estimated genome size, number of annotated genes and percentage of globally consumed kilocalories that they are responsible for

Species	Common name	Genome size (Mbp)	Number of annotated genes	Genome multiples^a^	Percentage kcal production [104]	Percentage genome captured^b^	Percentage transposon/repeat^c^	References
*Nelumbo nucifera*	Sacred lotus	929	26,685			86.5	57	[105]
*Beta vulgaris*	Sugar beet	758	27,421		1.2	74.8	63	[106]
*Solanum lycopersicum*	Tomato	900	34,727	36×	0.21	84.4	63.3	[107]
*Solanum tuberosum*	Potato	844	39,031	72×	1.51	86	62.2	[108]
*Solanum melongena*	Eggplant	1125	85,446	36×	0.07	74	70.4	[109]
*Capsicum annum*	Pepper	3480	34,903	36×	0.14	87.9	76.4	[110]
*Nicotiana benthamiana*	Tobacco	3000	ND			86.7	ND	[107]
*Vaccinium macrocarpon*	Cranberry	470	36,364		0.002	89.4	39.5^b^	[111]
*Actinidia chinensis*	Kiwifruit	758	39,040		0.005	81.3	36	[112]
*Coffea canephora*	Coffee	710	25,574	24×		80	50^b^	[113]
*Vitis vinifera*	Grape	475	30,434		0.36	102.5	41.4	[114]
*Populus trichocharpa*	Poplar	485	41,377			84.5	41	[115]
*Linum usitatissimum*	Flax	350	43,384			81	24.3^b^	[116]
*Ricinus communis*	Castor bean	320	31,237			100	50	[117]
*Manihot esculenta*	Cassava	742	30,666		2.05	70	36.9	[118]
*Hevea brasiliensis*	Rubber tree	2150	68,955			51	78	[119]
*Cucumis sativus*	Cucumber	367	26,682		0.04	70	24	[120]
*Cucumis melo*	Melon	450	27,427		0.04	83.3	19.7^b^	[121]
*Citrullus lanatus*	Watermelon	425	23,440		0.11	83.2	45.2	[122]
*Fragaria vesca*	Strawberry	240	34,809		0.009	95	22^b^	[123]
*Malus x domestica*	Apple	742	57,386	24×	0.22	81.3	38^b^	[124]
*Pyrus bretschneideri*	Pear	528	42,812		0.07	97.1	53.1	[125]
*Cannabis sativa*	Cannabis	818–843	ND			65.1	ND	[126]
*Humulus lupulus*	Hops	2570	41,228			80	34.7^b^	[127]
*Ziziphus jujuba*	Jujube	440	32,808			98.6	49.5	[128]
*Prunus persica*	Peach	265	27,582		0.06	84.8	18.6^b^	[129]
*Medicago truncatula*	Medicago	450	47,845	24×		54.6	31	[130]
*Cicer arietinum*	Chickpea	738	28,269	24×	0.29	73.8	49.4	[131]
*Lotus japonicus*	Lotus	472	30,799	24×		67	29.7^b^	[132]
*Glycine max*	Soybean	1100	46,430	48×	7.43	85	42^b^	[133]
*Cajanus cajan*	Pigeonpea	833	46,680	24×	0.11	72.7	51.67	[134]
*Phaseolus vulgaris*	Common bean	587	27,197	24×	0.754	80.6	45^b^	[135]
*Vigna radiata*	Mung bean	579	22,427	24×		80	50.1	[136]
*Lupinus angustifolius*	Lupin	1153	57,806			51.9	50	[137]
*Gossypium raimondii*	Cotton	630–880	37,505	72×	1.6	~100	61	[95]
*Gossypium hirsutum*	Cotton	2400	76,943	144×		~90	67.2	[96, 138]
*Theobroma cacao*	Chocolate	430	28,798	12×		76	41.8^b^	[139]
*Citrus x clementina*	Orange	367	25,376		0.17	82.1	45	[140]
*Carica papaya*	Papaya	372	28,629		0.02	73.8	41.9	[141]
*Brassica rapa*	Chinese cabbage	468–516	41,174	144×	1	59	39.5	[142]
*Brassica napus*	Oilseed rape	1130	101,040	288×	2.23	79	ND	[143]
*Brassica oleracea*	Vegetables	630	45,758	144×		85	38.8^b^	[128]
*Raphanus raphanistrum*	Wild radish	515	38,174			49.3	ND	[144]
*Phoenix dactylifera*	Date palm	658	28,890		0.08	60	ND	[145]
*Elaeis guineensis*	Oil palm	1800	34,802		5.09	85.3	50	[146]
*Musa acuminata*	Diploid banana	523	36,542	64×	0.41	90	32	[147]
*Oryza sativa*	Asian rice	389	37,544	32×	17.2	95	35	[51]
*Oryza glaberrima*	African rice	358	33,164	32×		88.3	34.3	[148]
*Hordeum vulgare*	Barley	5100	26,159	32×	3.23	37.3	84	[149]
*Triticum aestivum*	Wheat	17,000	124,201	96×	15.98	60	76.6	[150]
*Zea mays*	Maize	2500	32,540	64×	23.56	81.9	85	[151]
*Sorghum bicolor*	Sorghum	730	34,496	32×	1.99	89.7	61	[152]
*Setaria italica*	Foxtail millet	490	38,801	32×	1.01	86	46	[153]
*Eragrostis tef*	Tef	772	ND	64×		87	14^b^	[154]

^a^Reported whole-genome doublings from base of angiosperms as reported in [155] and inferred from phylogenetic position. ^b^As determined from the amount of sequence represented in the assembly compared to estimated genome size. For some species, these percentages were reported in the referenced articles, whereas for others we calculated the percentages using genome size estimates from articles in which sequences were published or from public databases. ^c^These percentages are likely to be underestimates. Abbreviations: ND No data/data not reported

### Genomic responses to polyploidy

**Fig. 1:**
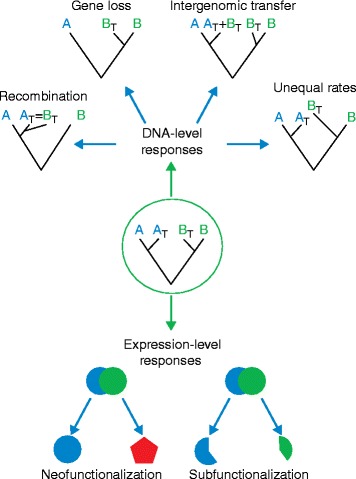
Processes and patterns of polyploidy in plants. Flowering plant genomes have an evolutionary history that includes multiple, lineage-specific, whole-genome doubling events. A model of a hypothetical allopolyploid genome derived from two progenitor diploid genomes (*A* and *B*) is shown in the figure. Hybridization and genome doubling set in motion short-term and long-term genomic processes, at the level of DNA sequence (*top*) and at the expression level (*bottom*). Young allopolyploids, which initially contain two genomes (*A*_*T*_ and *B*_*T*_) inherited from the parental genome donors, often display homoeologous recombination (‘gene conversion’), mutational loss and/or silencing of duplicated genes, intergenomic spread of TEs and differential rates of molecular evolution, as well as many different forms of biased or altered gene expression, including biased homoeolog expression and expression level dominance (not shown), and expression subfunctionalization (shown as partitioning of ancestral *blue* and *green* expression domains, *bottom left*) and neofunctionalization (novel *red* expression domain, *bottom right*)

**Fig. 2:**
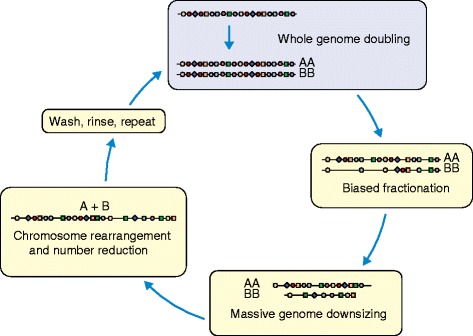
Episodic, cyclical episodes of polyploidy in plants. All plant lineages are paleopolyploid, differing only in the number of rounds of whole-genome doubling in their ancestry, and in the timing of these events. Long-term processes following whole-genome doubling include differential retention of duplicated genes (here, more *circles* than *squares* are lost), biased genome fractionation (shown as greater loss of the B genome), massive genome downsizing and reduction in the number of chromosomes

This enhanced appreciation of the history of plant genomes might make one ask why this history of repeated, episodic polyploidy was not recognized earlier. The answer to this question lies in the surprisingly varied spectrum of genomic responses to polyploidy [11–19], which range in timing from those accompanying the initial genome merging and doubling, to others operating over millions of years. As modeled in Fig. 1, the immediate responses to the formation of a polyploid (mostly allopolyploid) genome include DNA-level and expression-level responses. Examples of the DNA-level responses include reciprocal or non-reciprocal homoeologous exchange, mutational loss of duplicated genes, intersubgenomic spread of TEs (which can be activated by genome merging and polyploidization), and divergence in molecular evolutionary rates. Expression-level alterations accompanying or set in motion by polyploidy encompass a variety of forms of duplicate gene expression bias, and subfunctionalization and neofunctionalization of expression patterns. Long-term responses include genome-wide subfunctionalization and neofunctionalization [20–23] and massive genome structural rearrangements (Fig. 2). These structural rearrangements include reductions in chromosome numbers and the large-scale loss of repetitive sequences and duplicate genes [24–26]. Thus, new polyploid species, most of which have experienced multiple cycles of polyploidization, eventually experience massive loss of ‘redundant’ DNA and chromosome restructuring, and recurrent genome downsizing [26]. Thus, neopolyploid species ultimately become diploidized by mechanistically diverse processes, such that contemporary descendants increasingly behave cytogenetically as normal diploid species while harboring in their genomes the vestigial evidence of past WGD events.

### The fate of duplicated genes

An intriguing facet of this cyclical process of genome downsizing is that it may be non-random with respect to the fate of duplicate genes. Genes restored to single copy status often have broader expression domains and higher expression levels than those retained in duplicate; they are also enriched for essential housekeeping functions, chloroplast-related functions, and functions in DNA replication and repair [27]. Although much remains to be learned in this active area of investigation, the evolutionary forces underlying the fate of duplicated genes include those emerging from the selective demands of stoichiometry during protein complex assembly, or the necessity of maintaining balanced protein interactions, and other possibilities involving higher-order interactions of protein function within biological networks [27–30]. For example, genes encoding proteins that function as monomers with few interacting protein partners or that function in downstream parts of biological pathways are expected to experience fewer functional constraints than those encoding proteins that have numerous protein–protein interactions, function as parts of protein complexes, are highly connected in biological networks, or function in upstream parts of pathways with multiple downstream epistatic effects.

A second, fascinating aspect of this ‘duplicate gene diploidization’ phenomenon is that the origin of the retained genes, when compared with the origin of the genes that are lost, may be strikingly non-random with respect to the two donor diploid genomes. This ‘biased fractionation’, which has now been detected in both monocots and eudicots [24, 31, 32], is an utterly unexpected process that has even been reported to have occurred after allopolyploid events that trace to the start of the Tertiary [33]. In this example, differential retention of ancestral genomes involved in a 60-million-year-old polyploidization event in the ancestry of cotton remains evident in modern cotton diploid species. The evolutionary drivers of biased fractionation are incompletely understood and might be different in different taxa, but are likely to involve, among other factors, the interplay between selection and adjacency of genes to TEs that might have a repressive effect on gene expression (and thereby render these genes more ‘expendable’ than their homoeologs) [25, 33].

## Transposable elements and genome size variation

“The history of the earth is recorded in the layers of its crust; the history of all organisms is inscribed in the chromosomes” (H. Kihara [34]).

On completion of the first plant genome, that of *Arabidopsis thaliana*, it was already clear that even the ‘simplest’ of plant genomes is a mosaic derived from multiple rounds of polyploidy events [35]. Since then, dozens of additional genomes have been sequenced, including those of most major crop plants (Table 1) [36]. Much like ancient palimpsests, sequenced genomes metaphorically reveal, at the sequence level, the reused manuscript pages from previous authors, or, as described above and in Fig. 2, the nested remnants of previous WGD events. Many of these surviving duplicated regions regulate gene activity and function, so genomic archaeology and even paleontology are essential to reveal the *scriptio inferior*, the history and hidden messages contained in genome sequences.

One revelation emanating from these studies concerns the genesis of the extraordinary variation in plant genome sizes [37–39]. WGD events are an obvious route to genome expansion, but many ‘diploid’ species have enormous genomes. For example, the barley genome is ~11.5 times larger than that of another cereal, rice (5.1 Gbp and 0.43 Gbp, respectively). In addition to polyploidy, genome size can saltationally increase owing to rapid proliferation of TEs [40], notwithstanding mechanisms for removal of these elements, such as unequal and illegitimate recombination [41]. Lineage-specific amplification, and potentially deletion, of TEs is common in plants, even among closely related species, such as between subspecies of domesticated rice, *Oryza sativa* subsp. *indica* and subsp. *japonica* [42]. Within the same genus, *O. australiensis* has a genome that is more than twice the size of that of *O. sativa*, mostly as a result of the addition of ~400 Mbp of DNA in the past few million years by three individual retrotransposable element families [43]. A clade of Australian cotton (*Gossypium*) diploid species have a nearly three-fold larger genome than those of the American diploid clade, owing to lineage-specific proliferation and deletion of different families of TEs [44, 45]. These examples highlight that the majority of variation in plant genome size reflects the dynamics of TE proliferation and clearance, superimposed on a history of WGD [38, 39]. Although this pattern is now known, the underlying causes of TE proliferation are far less well understood. Why are some TEs amplified in some genomes but not in others, even when they are present? For instance, the elements that resulted in doubling of the *O. australiensis* genome are present in all other *Oryza* lineages but have remained largely inactive, except for the TE *Gran3* of *O. granulata*, which caused a ~200 Mbp retroelement burst of activity approximately 2 million years ago in this species. *Gran3* is related to the *Wallabi* TE of *O. australiensis* [43, 46]. Are there certain ecological conditions that govern or trigger these TE proliferation events?

### Constancy of genic content yet enormous variation in genome size

Despite their extraordinary range in size, from the tiny 60 Mbp genome of *Genlisea aurea* to the enormous >150 Gbp genome of *Paris japonica*, plant genomes have comparatively little variation in gene content [47]. This fact reflects the combined effects of TE proliferation, which dwarfs the effects of tandem or dispersed gene duplication in increasing genomic DNA content, and the process of long-term genomic fractionation, which is associated with loss of most gene duplications following WGD (Fig. 2). TEs have been implicated as important factors in gene regulation and adaptation, particularly with gene content being fairly consistent across plants and the rapid accumulation and removal of TEs [48–50].

### Implications for genome assembly and interpretation

Most of the insights about plant genomes were enabled by cytogenetics, molecular genetics and, now, high-throughput sequencing technology. In fact, the majority of our food and fiber crops have at least one genome assembly in the public domain (Table 1). However, the quality of these genome assemblies varies considerably, reflecting a transition from map-based Sanger sequencing (e.g., [35, 51]) to second-generation, low-cost, short-read, whole-genome shotgun sequencing that generally yields ‘gene space’ assemblies. The complexities of genome sequencing in plants with large genomes or in those that have experienced recent polyploidy have often been quite vexing because of the high sequence similarity among recently merged or doubled genomes. This challenge has been particularly true for large allopolyploid genomes, such as that of wheat (~15 Gbp), *Triticum aestivum*, for which a high-quality reference genome has yet to be released. The preponderance of highly similar repetitive elements in these genomes means that these are often excluded from whole-genome assemblies. This exclusion is an important consideration not just for the sake of genome completeness per se, but also because many of these repeats are the primary targets of epigenetic/chromatin remodeling pathways that often affect the expression or structure of genes [39, 52]. Third-generation, long-read (5 to >40 kbp read length) sequencing technologies from platforms such as Pacific BioSciences [53] and Oxford Nanopore [54] are bringing us to a future of high-quality, gap-free genome sequences, which are necessary to more fully understand genome structure and function. Within the next two to three years we anticipate that most of the assemblies listed in Table 1 will be upgraded, or even replaced, using these new technologies.

### Resequencing and pangenomes

Reference genome sequences are but snapshots of single genomes frozen in time. However, plants continue to evolve, adapt and diversify, so the genetic variation revealed in a single genome sequence fails to adequately represent the variation present within a species. Reference genomes have become highly useful as templates for ‘mapping’ resequencing data from additional accessions, which has led to insights into the structure and history of genetic variation within a crop plant or other species [55]. Resequencing, however, is limited by the inefficiency of mapping short reads in variable genomes, particularly in species with abundant genomic variation and TE activity. Accordingly, variants larger than single nucleotides or small insertions or deletions (indels) are often not captured in resequencing datasets, so many intergenic sequences that might be important in gene regulation are missed [56]. Moreover, the effect of TEs on presence–absence variation and on the evolution of new genes (with Pack-MULE [57] or TRIM [58] TEs being examples of the latter effect) within a genus or species might not be captured in a single genome sequence. Pantranscriptomes [59] and pangenomes have emerged as tools to effectively capture this additional layer of variation. This strategy entails sequencing multiple genomes within a species, as in soybean (*Glycine soja*) [60] or maize [61, 62], or even an entire genus (e.g., *Oryza* [63]), such that diverged and rearranged sequences can be mapped and analyzed. High-quality pangenome references capture natural variation and rare variants that are important for the identification of genes or regions associated with adaptation to environmental conditions and for crop improvement. DivSeek [64] and the Global Crop Diversity Trust [65] are examples of organizations seeking to coordinate resequencing efforts of entire germplasm collections. The International Rice Research Institute (IRRI), the Chinese Academy of Sciences (CAS) and the Beijing Genomics Institute (BGI) also recently coordinated the resequencing of ~3000 diverse rice accessions [64]. Notably, only a single high-quality reference genome exists for Asian cultivated rice, and so a large proportion of the resequencing data are unmappable. This observation demonstrates the need for high-quality pangenome reference sets, not just for rice but for the majority of crop plants.

## Small RNAs, mediators of interactions in duplicated genomes

**Fig. 3:**
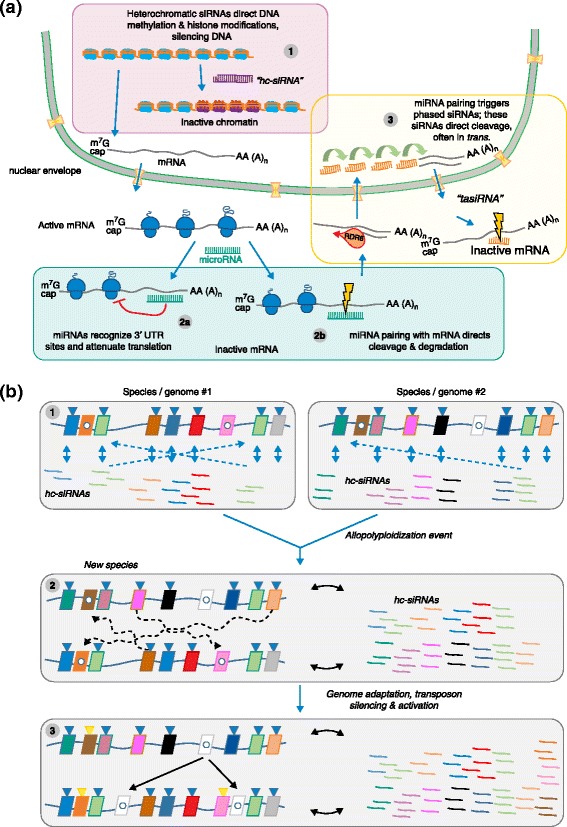
The role of small RNAs in plant genome evolution. **a** Plant genomes are rich sources of small RNAs, which are predominantly the products of three major pathways. (1) Heterochromatic siRNAs (hc-siRNAs) are 24-nucleotide products of the activity of the plant-specific Pol IV and Pol V pathways; hc-siRNAs derive from heterochromatic regions and target those regions for reinforcement of silencing chromatin marks. (2) MicroRNAs (miRNAs) are ~21-nucleotide or 22-nucleotide products of processed long noncoding mRNAs that function to suppress target mRNAs either by (2a) blocking translation, or (2b) directing Argonaute-mediated slicing of targets. Plant miRNAs typically function by directing slicing. (3) Some miRNAs, typically 22 nucleotides long, trigger the production of secondary siRNAs, which are products of DCL4 and are 21 nucleotide long, from their target mRNAs. This pathway requires RDR6, and some resulting siRNAs can act in *trans* to slice additional targets; hence their name of *trans*-acting siRNAs (tasiRNAs). **b** hc-siRNAs are typically derived from TEs, the predominant component of inactive chromatin in plant genomes. Transposons (colored parallelograms in 1–3) can be silenced (*blue triangles*) as a result of RNA-directed DNA methylation mediated by hc-siRNAs derived from those elements (*thin blue arrows*). Some transposons can escape DNA methylation and silencing (*white dots*), to later remobilize. Transposons can be additionally silenced by hc-siRNAs functioning in *trans* from related TEs (*dashed lines*). After allopolyploid individuals form (2), the genomic context changes for TEs from the progenitor genomes, and these elements can be silenced by hc-sRNAs derived from sequence-similar TEs residing in the added genome (*dashed, curvy lines*). After this period of adaptation (3), some TEs can be newly silenced (*gold triangles*), whereas a few might remain unsilenced and active, and might amplify into the added genome (*thin black arrows*). *UTR* untranslated region

Small RNAs are important modulators and mitigating factors of the effects of genome duplications and TE-driven genomic expansion on genome architecture. Several recent reviews have highlighted the diversity of small RNAs and their ability to function in *trans* to direct or communicate their silencing effects across members of gene or TE families [66, 67]. These molecules are classified into three major classes that have distinct roles in gene or TE regulation: (1) microRNAs (miRNAs) that are derived from mRNA precursors produced by the processing activity of Dicer-like 1 (DCL1) and that function in post-transcriptional control of target mRNAs; (2) secondary small interfering RNAs (siRNAs) that are also processed from mRNAs by a Dicer enzyme (DCL4 or DCL5) and typically have a phased configuration (phasiRNAs), which can function against other mRNAs as *trans*-acting siRNAs (tasiRNAs); and (3) heterochromatic siRNAs (hc-siRNAs), which derive from precursors transcribed by plant-specific Pol IV and Pol V enzymes and are processed by yet another Dicer enzyme (DCL3) (Fig. 3). Each of these three classes of small RNA has a suppressive activity: 21-nucleotide or 22-nucleotide mature miRNAs reduce protein levels, typically by reducing the expression of their target transcripts, in diverse pathways often related to development or stress responses; 21-nucleotide or 22-nucleotide tasiRNAs or phasiRNAs have roles that are similar to those of miRNAs or have yet uncharacterized roles; and 24-nucleotide hc-siRNAs function as ‘guardians of the genome’, providing stable, multigenerational protection against invasive transposons. Extensive analysis of sequenced plant genomes has shown each of these classes of small RNAs has distinct evolutionary paths and influences on genome structure that reflect their functional roles [66, 67].

### Small RNA responses to WGD events and TE proliferation

Immediately following WGD events, duplicated genes and TEs are expected to experience a relaxation of selection that is due to functional redundancy at the time of duplication. The implications of this relaxed selection vary among genes and TEs, and among the types of small RNAs that have regulatory roles. Mature miRNAs are processed from transcripts of precursor genes (*MIRNAs*) that are influenced by the same events occurring at the whole-genome scale that shape gene and genome evolution, including polyploidy and other mechanisms underlying gene duplication [68]. Like for protein-coding genes, the emergence of lineage-specific miRNAs is fairly common, although a core set of well-conserved miRNAs also exists [69]. In soybean, duplicated (i.e., multi-copy) *MIRNAs* were retained at a higher rate than single-copy *MIRNA*s, with this higher rate resulting from functional constraints and genomic duplication events local to the *MIRNAs* [68]. The evolutionary basis for this finding is unclear, but this observation could reflect the functional importance of miRNA dosage when target genes are duplicated. As a consequence of genomic duplications, some well-conserved miRNAs are found at increased copy numbers in species that underwent recent polyploidy. For example, there are 22 copies of the miR165/166 family found in the recently duplicated soybean genome, whereas nine copies are observed in the *Arabidopsis* genome. This high dosage is not yet known to have functional relevance, but the evolutionary decay of duplicated *MIRNA* genes might be slowed because the most functionally relevant portion of the mRNA precursor of a miRNA is presumably the hairpin structure, which is just a few hundred nucleotides in length. The importance of precursor components 5’ and 3’ of this stem-loop are, however, still poorly characterized in plants. Strong selection for sequence conservation in miRNAs in regions other than their promoter elements is likely to be largely limited to nucleotides within the hairpin that are needed for processing, plus those in the mature miRNA that are required for successful targeting. The net effect of this limited selection for conservation is that miRNAs might have fewer positions at which mutations would be functionally equivalent to nonsense or missense mutations than protein-coding genes; hence, miRNAs might have a longer half-life than protein-coding genes following WGD events.

Like miRNAs, phasiRNAs are generated from mRNAs, and thus their precursors (*PHAS* loci) are duplicated or lost through the many processes that also affect deletion and replication of other genomic regions. As far as we know, the important functionally constrained nucleotides in phasiRNA mRNA precursors include promoter elements, the miRNA target site and the typically few phasiRNAs that have important targets. To date, the targets of phasiRNA derived from long, non-coding RNAs are largely unknown, although in a few cases they have been well-described, such as the 21-nucleotide “tasiARF” in *TAS3* [70]; in the case of TAS3, it is just one or two of many 21-nucleotide phasiRNAs from the locus that is believed to be functional. Thus, as *MIRNA* genes, *PHAS* genes might be slower to pseudogenize than protein-coding genes, and, therefore, their retention time after polyploidization might be longer than that of protein-coding duplicates. The appearance of novel *PHAS* loci has also been recorded. In the *Medicago* and soybean genomes, for example, non-conserved, flower-enriched or anther-enriched *PHAS* loci exist. Some of these loci seem to target transposons, perhaps as a mechanism to suppress TE activity during reproduction [71, 72]. However, most changes in *PHAS* loci are likely to be spontaneous events, independent of polyploidy events.

In comparison to miRNAs and phasiRNAs, hc-siRNAs, as derivatives of TEs, are subject to numerous stochastic and selective evolutionary forces that shape genomes, and are likely to be critical in the modulation or mitigation of the effects of WGD events. hc-siRNAs function as primary defenses against ‘invasive’ TEs acting as a sort of “vaccine” against deleterious elements. Even so, they are derived directly from TEs through the specialized pathway of RNA-directed DNA methylation (RdDM), produced via TE precursors transcribed as RNAs long enough to generate a hc-siRNA, but too short to encode a functional TE [73]. Their derivation from TEs allows them to provide direct genomic defenses against TEs, but their transcription by Pol IV and Pol V and their retention in the nucleus prevents their translation into invasive TEs, thereby avoiding any potential adverse effects. Evolutionary analysis indicates that the RdDM pathway is fairly ancient in plants, with components dating to before the divergence of mosses, in which 23-nucleotide siRNAs direct RdDM [74]; later, in gymnosperms, hc-siRNAs achieved their ‘modern’ size of 24 nucleotides [75], with substantial diversification in the components of the pathway occurring throughout the evolution of gymnosperms and later in angiosperms [76]. Perhaps this elaboration of the machinery for silencing facilitated polyploidization events by providing an effective mechanism for suppressing TE activity, which otherwise might have been more rampant, and hence disruptive, following genomic mergers.

hc-siRNAs are believed to function in *trans* to direct silencing at related elements via sequence homology [77], although this hypothesis has not been thoroughly tested and we do not have a good idea of the degree of homology that is required for such *trans* activity. Nevertheless, we can speculate that novel and important interactions occur between the two suites of distinct hc-siRNAs and TEs that become suddenly merged within the same genome during allopolyploidization events (Fig. 3b). One possible outcome of this form of biological reunion is that hc-siRNAs function to suppress TEs both in *cis* and in *trans*, and hence that TEs are no more likely to mobilize than in the originally separate genomes. Alternatively, interspecific hybridization and WGD events might be accompanied by a burst of TE proliferation, perhaps as a direct consequence of a destabilized or altered population of hc-siRNAs and their influence on DNA methylation or chromatin states (Fig. 3b) [78–80]. Perhaps TEs escape silencing by flying under the genomic surveillance radar [81, 82], and thereby proliferate and invade new genomic space, which would have multiple effects on genomic structure and gene evolution [39]. These effects would be particularly important in reproductive tissues, in which TE silencing is less effective; this hypothesis is supported by growing evidence [83]. The TE complement of plant genomes usually consists of various TE families that massively amplified through ancient bursts of proliferation (as in *O. australiensis* [43]), and many of these genomic explosions are likely to represent a ‘failure of the vaccine’ — an escape from detection and suppression of TEs. The proximal trigger of bursts of TE proliferation is not understood, but could involve mechanisms that disable defenses via suppression of silencing or ephemeral developmental periods during which RdDM is less active in germline cells, or perhaps during the formation of zygotes. For example, asymmetric contributions of the maternal and paternal gametes, including siRNAs or modifiers of silencing processes [84–87], could differentially influence the TEs in the resulting zygote, potentially allowing some TEs to proliferate.

## Conclusions and future perspectives

The genomes of the approximately 300,000 species of flowering plants exhibit extraordinary variation in size and their complement of genomic elements. This variation is the outcome of temporally dynamic and phylogenetically variable, even idiosyncratic, interplay among processes set in motion by episodes of polyploidy, TE proliferation and regulatory events mediated by small RNAs. These events are all molded by even more complex biotic and abiotic interactions between the organisms and their environments. What are the broad implications of this new and improved view of the origin of the modern angiosperm genome architecture? This perspective might be fundamental to much of plant biology, as many different processes, be they metabolic, physiological or ecological, are specified by the size and functional diversification of contemporary multigene family structures, gene expression patterns and the systems biology context of various genomic elements. These processes all operate within a genomic milieu of TEs and small RNAs that partly originate from the survivors of past ‘wash–rinse–repeat’ cycles of polyploidization followed by non-random and incomplete diploidization. These endpoints, having been shaped by diverse selective and, presumably, neutral forces, have generated the genic and genomic architecture that underlies all plant phenotypes, be they physiological, ecological or morphological [8, 27, 88, 89]. An exciting area for future research is the exploration of the connections between the short-term and long-term responses to WGD and the interconnections of these responses with TE proliferation and small RNA evolution, both in terms of molecular mechanisms and implications for natural selection. This challenge will necessitate a multidisciplinary, integrative approach and biological investigation of multiple model allopolyploid systems and natural ecological settings. The use of experimentally tractable systems, including synthetic polyploids and their natural relatives, to explore the interconnections between the phenomena we have highlighted and the evolutionary ecology of specific lineages is an exciting prospect. Now that large-scale ‘omics’ datasets of genomes, transcriptomes, epigenomes, etc. are increasingly becoming available within or across species, trans-disciplinary teams will be more able to understand plant responses to varying environments and long-term adaptation. These studies will contribute to understanding basic biological processes and are a prelude to engineering these process for the betterment of humankind.

This fundamental genomic understanding is likely to be valuable for crop improvement. Oliver et al. [50] tabulated 65 examples of TE insertions in regulatory or coding sequences that affect a wide range of phenotypic traits, such as skin color in grape [90] and anthocyanin accumulation in blood orange [91]. The most famous example involving a TE insertion and crop productivity is perhaps the insertion of the *Hopskotch* TE in the far-upstream regulatory region of *tb1* in maize, which enhanced *tb1* expression and promoted the typical architecture of the maize plant relative to that of its progenitor, teosinte [92]. Gene and genome doubling have also been shown to be important in agriculture, as summarized by Olsen and Wendel [93]. Examples of this importance are seen in major grains such as wheat and rice, as well as in other crop plants such as tomato and sunflower. In addition to cases in which known TE insertions or duplicated genes have been shown to affect crop plant traits, the more general importance of these events has been appreciated, even when the specific lesions are not understood. For example, in the most important species of cotton (*G. hirsutum*), which is allopolyploid, the two co-resident genomes have intermingled and contribute unequally to fiber quality and yield [94–98]. In maize, large genotype–phenotype association studies have shown that modern paralogs descended from the most recent WGD are ~50 % more likely to be associated with functional and phenotypic variation than singleton genes, which highlights the importance of genome-wide neofunctionalization in generating new variation [99]. As is the case for TEs and WGD events, diversification, evolution and selection of small RNAs are potentially important processes in crop plants, including rice [49, 64] and cotton [99]. In cotton, only one of two homoeologs of an mRNA that encodes a MYB transcription factor underwent preferential degradation during cotton fiber development, which makes this case particularly illustrative of a direct link between a recent WGD event and miRNA behavior. Further work is needed to understand the interplay between TE proliferation, insertion/retention bias in polyploid plants and small RNA biology, and how to harness this biology to enhance traits of agronomic importance.

Genome sequences also provide many insights into the paleogenomic record of plant life, but, as with paleontology, not all features fossilize equally well and the record is incomplete.

The majority of plant genome sequences are from crop plants. Crop genome sequences anchor large commodity-based communities around a single resource that can be leveraged in numerous directions for crop improvement and basic discoveries. Reference genomes can now be used by germplasm banks worldwide. These banks contain domesticated crop relatives that are adapted to grow under varied environmental conditions and that harbor untapped reservoirs of traits that can be used for crop improvement. How can one exploit the knowledge of genomic evolutionary processes to tap into these resources and thereby create new traits that will empower the next green revolution? An initial step would be to genotype gene bank collections [100]. A landmark example of this approach was the recent resequencing of 3000 cultivated rice accessions representative of two large rice gene banks, from which more than 18.9 million new single nucleotide polymorphisms were discovered [64, 101]. Another example is the *Seeds of Discovery* project at International Maize and Wheat Improvement Center (CYMMIT) in Mexico, where 27,500 and 30,000 maize and wheat accessions, respectively, have been genotyped and are being phenotyped [102]. As discussed above, pangenomic resources will be needed to more efficiently capture the variation from these resequencing and genotyping projects. Such data can then be integrated into genomic selection breeding programs to drive the generation of tomorrow’s crops.

The importance of this agenda is difficult to overstate. The United Nations projects that the world population will exceed 9.7 billion by 2050, with the majority of growth coming from Africa and Asia [103]. One of the biggest challenges we face is how to feed an additional ~2.4 billion people in less than 35 years in a sustainable and environmentally responsible way. By unraveling the history of plant genomes and their genomic ecosystems we can begin to understand how natural selection shaped genomes in time and space to adapt to different environmental conditions. Genomic information will allow us to develop high yielding and sustainable genotypic combinations that are more efficient in the use of nutrients and water, resistant to insects and pathogens, and more nutritious.

## Abbreviations

EST: Expressed sequence tag
hc-siRNA: Heterochromatic siRNA
phasiRNA: Phased, secondary siRNA
RdDM: RNA-directed DNA methylation
siRNA: Small interfering RNA
tasiRNA: *Trans*-acting siRNA
TE: Transposable element
WGD: Whole-genome doubling
